# Design and evaluation of a LIS-based autoverification system for coagulation assays in a core clinical laboratory

**DOI:** 10.1186/s12911-019-0848-2

**Published:** 2019-07-03

**Authors:** Zhongqing Wang, Cheng Peng, Hui Kang, Xia Fan, Runqing Mu, Liping Zhou, Miao He, Bo Qu

**Affiliations:** 1 Department of Health Statistics, School of Public Health, China Medical University, 77 Puhe Road, Shenyang North New Area, Shenyang, 110122 China; 2 Department of Information Center, The First Affiliated Hospital of China Medical University, Shenyang, China; 3 Key Lens Laboratory of Liaoning Province, Department of Ophthalmology, The Fourth Affiliated Hospital of China Medical University, Shenyang, China; 4 Department of Clinical Laboratory, The First Affiliated Hospital of China Medical University, Shenyang, China

**Keywords:** Laboratory information systems, Medical safety, Autoverification, Coagulation, Turnaround time

## Abstract

**Background:**

The autoverification system for coagulation consists of a series of rules that allow normal data to be released without manual verification. With new advances in medical informatics, the laboratory information system (LIS) has growing potential for the autoverification, allowing rapid and accurate verification of clinical laboratory tests. The purpose of the study is to develop and evaluate a LIS-based autoverification system for validation and efficiency.

**Methods:**

Autoverification decision rules, including quality control, analytical error flag, critical value, limited range check, delta check and logical check, as well as patient’s historical information, were integrated into the LIS. Autoverification limited range was constructed based on 5 and 95% percentiles. The four most commonly used coagulation assays, prothrombin time (PT), activated partial thromboplastin time (APTT), thrombin time (TT), and fibrinogen (FBG), were followed by the autoverification protocols. The validation was assessed by the autoverification passing rate, the true-positive cases, the true-negative cases, the false-positive cases, the false-negative cases, the sensitivity and the specificity; the efficiency was evaluated in the turnaround time (TAT).

**Results:**

A total of 157,079 historical test results of coagulation profiles from January 2016 to December 2016 were collected to determine the distribution intervals. The autoverification passing rate was 77.11% (29,165/37,821) based on historical patient data. In the initial test of the autoverification version in June 2017, the overall autoverification passing rate for the whole sample was 78.75% (11,257/14,295), with 892 true-positive cases, 11,257 true-negative cases, 2146 false-positive cases, no false-negative cases, sensitivity of 100% and specificity of 83.99%. After formal implementation of the autoverification system for 6 months, 83,699 samples were assessed. The average overall autoverification passing rate for the whole sample was 78.86% and the 95% confidence interval (CI) of the passing rate was [78.25, 79.59%]. TAT was reduced from 126 min to 101 min, which was statistically significant (*P* < 0.001, Mann-Whitney U test).

**Conclusions:**

The autoverification system for coagulation assays based on LIS can halt the samples with abnormal values for manual verification, guarantee medical safety, minimize the requirements for manual work, shorten TAT and raise working efficiency.

## Background

Following the analytical phase, a large number of manual verifications are performed in clinical laboratories to detect possible errors before the results are released to the electronic health records, which is time-consuming [1]. A significant answer for this issue may be autoverification, which is a set of well-designed rules with an aim to stop the samples with abnormal values for manual verification and at the same time permit those with normal values to be released without manual intervention [2]. Previous reports have demonstrated that autoverification can ensure medical safety [3], shorten turnaround time (TAT) [2–5], reduce labour requirements [2–4], improve operational efficiency [2, 4–6] and minimize error rate [2], as well as enable laboratory technologists to devote more attention to test results with greater potential error [2].

Until recently, those autoverification systems were commonly developed via third party commercial software or middleware, which are costly, and the autoverification decision rules were proprietary, such that no revision could be made according to the user’s requirements [7–10]. In addition, they cannot connect with the hospital information system (HIS) and obtain comprehensive clinical data, such as patient’s history and clinical diagnosis. With the progress of laboratory automation, it is challenging to achieve interconnection and intercommunication between analytical instruments and the laboratory information system (LIS) to design within-laboratory autoverification systems independent of any commercial software.

Coagulation assays are essential for assessment of patients requiring acute care [11], patients undergoing anticoagulant therapy [12], thrombolytic therapy [13] and pregnancy [14], as well as monitoring of disseminated intravascular coagulation [15]. Currently, in many laboratories the coagulation assays are still released by manual review, verification and release, and reports about autoverification in coagulation are scarce [16]. The four most routinely used coagulation assays in our laboratory, namely, prothrombin time (PT), activated partial thromboplastin time (APTT), thrombin time (TT) and fibrinogen (FBG), are commonly prescribed together. There is therefore an urgent need, but it is still a challenge to establish autoverification decision rules in coagulation, including quality control (QC) check, instrument error flags, critical values, limited range check, delta check, logical rules and patient’s historical data, with reference to the Autoverification of Clinical Laboratory Test Result; Approved Guideline (AUTO 10-A) [17]. This research extends our knowledge to establish a within-laboratory autoverification system for coagulation assays based on LIS to ensure medical safety and shorten the turnaround time (TAT). Moreover, it is the first study based on clinical large-scale data to evaluate the validation and efficiency of autoverification in terms of specificity and efficiency.

## Methods

### Setting

The study was conducted in the core clinical laboratory of the First Affiliated Hospital of China Medical University, which processes approximately 3,500,000 various laboratory samples per year. The HIS and the LIS were both provided by Donghua Co-creation software (Beijing, Donghua, China). The autoverification decision rules were converted into computer languages to integrate into the HIS. In the process of autoverification, the values that passed the autoverification protocols were marked in green, and those that failed were flagged in red. When abnormal results were held up, a quick attentive manual verification or re-analysis was needed. If the results were considered to be critical values, they were quickly released to the HIS, and the clinicians were alerted to the abnormalities by a call.

### Data collection

Coagulation profiles from outpatients, inpatients and patients from the physical examination center of our hospital were collected for analysis. Historical values of 157,097 samples between January 2016 and December 2016 were used to determine the 5 and 95% percentiles of PT, APTT, TT and FBG. A total of 37,821 historical records released by manual verification from January 2017 to March 2017 were obtained to test the passing rate of autoverification protocols. In the test version phase, validation of the autoverification protocols was determined by 28,244 samples between May 2017 and June 2017. After the protocols were formally implemented online, 83,699 samples between July 2017 and December 2017 were taken to evaluate the single test autoverification passing rate and the overall autoverification passing rate.

### Analytical instruments and reagents

All tests were measured using STA-R Evolution Analyzer (Stago Diagnostica, Brussels, Belgium). Reagents of STA-PTT (APTT), STA-Neoplastin R (PT), STA-TT (TT), STA-Fibrinogen (FBG) and controls were also provided by Stago.

### Autoverification decision making

**Fig. 1:**
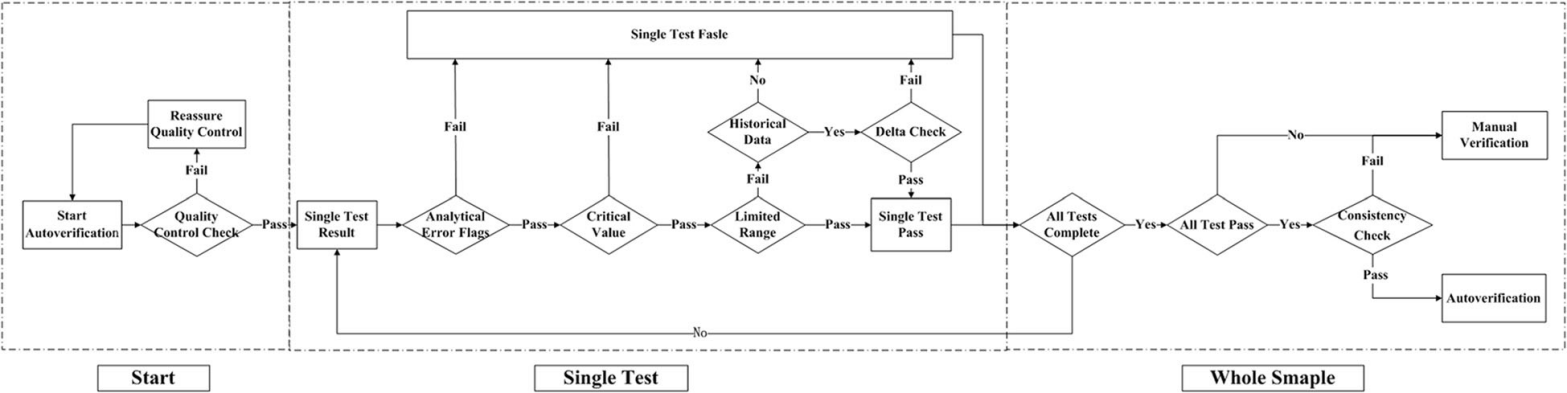
Autoverification decision-making tree. Autoverification procedures for single tests and whole samples in the order of quality control check, analytical error flags, critical values, limited range check, delta check, logical rules and patient’s clinical diagnosis

The autoverification system was designed with reference to Auto 10-A guideline [17]. A technologist-augmented decision-making tree was processed, and if any of the rules was violated, the result would be held for manual verification, as shown in Fig. 1. The autoverification steps consisted of quality control (QC) check, analytical error flags, critical values, limited range check, delta check and logical rules, as well as the patient’s clinical diagnosis. The autoverification protocols run the single test successively until the 4 items of the whole sample were finished. If all 4 items passed the autoverification, the logical rules were run for further verification; if any of the steps violated the rules, manual verification was required. Because the LIS is connected to the HIS in our hospital, any result that passed autoverification was directly released to the patients’ medical records without manual verification.

#### Quality control check

QC check is routinely performed in our laboratory, transmitting from the analyzer to the LIS according to Westgard quality control multi-rules [18]. The QC check ensured that each test was within the appropriate range before running the autoverification protocols. If there were no QC results within 24 h or if any QC rule was violated, the program halted all samples being autoverified.

#### Analytical error flags

If any instrument analytical flag was sent to the LIS along with testing problems, such as the two-dimensional barcode, reagents, and samples, as well as reagent crystallization or sample clots forming, the results were held for later manual verification.

#### Critical value

The critical values that may threaten the patient’s life and require urgent medical treatment were determined by laboratory technologists and clinicians. The critical values used for autoverification were as follows: PT ≤  9.00 s or  ≥  70.00 s, APTT ≤ 15.00 s or  ≥  100.00 s, TT >  150.00 s and FBG <  1.00 g/L, as shown in Table 2. If critical values were detected, they were manually verified by a skilled laboratory specialist, repeated, and transferred to a critical value list for an immediate phone call to clinicians. The results within the range ran the following limited range check and delta check.

#### Limited range check

The limited range is a filter to verify if the result is within the extreme reference intervals specified for the assays in the LIS. The clinical reference intervals used in our laboratory were 11.00–14.30 s for PT, 32.00–43.00 s for APTT, 14.00–21.00 s for TT and 2.00–4.00 g/L for FBG. In clinical practice, PT and TT values greater than 3.00 s and APTT value greater than 100.00 s are considered pathological. However, due to biological variation, clinical reference intervals are not always suitable for autoverification; we therefore analyzed the distribution of 5 and 95% percentiles based on the historical large-scale data from January 2016 to December 2016. In the current study, after discussing with local clinicians, the limited range was determined by a combination of clinical reference intervals, critical value and 5 and 95% percentiles. The results that were within the limited range continued to the next step.

#### Delta check

It is widely accepted that test results with historical values should be confirmed by a delta check [19, 20]. In our study, delta check was used for the results with historical data and failing the limited check. The delta check was performed to compare present test results with previous results and to identify variations beyond patient’s expected values, such as changes in a patient’s clinical condition or specimens with presence of clots [20–24]. Delta check can be performed using various methods, including the delta difference, delta percent change, rate difference, and rate percent difference [22, 25, 26]. In the present study, delta differences were calculated as the difference value between the present results and the previous results. Meanwhile, different tests correspond to different delta differences and time intervals due to biological and pathological functions of coagulation assays. The delta check rules were as follows: PT <  10.00 s and the time interval < 10 days; APTT < 10.00 s and the time interval < 7 days; FBG < 1.00 g/L and the time interval < 3 days. However, if the patient was undergoing anticoagulant therapy, the time interval was < 30 days, and the international normalized ratio (INR) was between 2 and 3.

#### Logical rules

Based on clinical and practical diagnostic criteria, a few tests may have some necessary logical interrelationships between each testing item, e.g., prolonged PT and APTT associated with a decreased FBG. Logical rules were applied with the condition that all single tests passed the QC check, analytical error flags, critical value, limited range check and delta check. If any result violated the logical rules, the autoverification protocols were stopped, and the results were reviewed manually before being sent to the HIS. In logical check, even though the single results were within the limited range, the reports were held for manual verification when PT ≥ 14.00 s or APTT ≥ 43.00 s and FBG ≤ 2.00 g/L, PT ≥ 14.00 s and APTT ≥43.00 s, or APTT ≤43.00 s, PT ≤ 14.00 s and TT ≥ 21.00 s occurred. By logical rules, a presence of clot was avoided.

#### Patient’s clinical diagnosis and historical data

Because it is of great significance for us to design precise and customized rules that work for inpatients and outpatients, the patient’s specific information, such as gender, age, clinical presentation, medical treatment history, drug history, pregnancy and other conditions that may affect the results of coagulation assays should be comprehensively considered. Adjustment of the limited range was done according to clinical diagnosis and historical data. For instance, for patients taking warfarin, the limited range was PT > 40.00 s, and the allowable ranges for INR were as follows: 1.50–2.50 before non-hip surgery, 2.00–3.00 before hip surgery, 2.00–3.00 for deep vein thrombosis, 2.00–4.00 for treatment of pulmonary infarction, 3.00–4.00 for prevention of arterial thrombosis, and 3.00–4.00 for valve prosthesis surgery. For patients receiving heparin treatment, the limited range was APPT > 90.00 s. If the patient is undergoing anticoagulation therapy, even though the test result of PT was within the routine limited range rather than within the customized limited range, the report would be stopped for manual autoverification and would not be sent to the HIS. For late pregnancy, the upper limit intervals for PT and APTT were decreased by 10%, and the lower limited range for FBG was increased by 10% [14]. For patients undergoing thrombolytic therapy, the limited range for FBG was defined as 1.20 g/L – 4.00 g/L.

### Assessment of the validation of the autoverification system

#### Assessment of the historical records test

According to CLSI Auto-10 A, all rules and settings must be validated and thoroughly tested before the autoverification protocols were implemented in a real-life clinical environment. First, we used historical clinical laboratory records stored in the LIS to verify the validation. To test if the program would screen the results outside the autoverification decision rules, special abnormal cases were chosen to test whether or not the autoverification program could be held up. Second, the autoverification passing rate of historical records in the LIS from January 2017 to March 2017 was determined to test if the autoverification protocols followed the rules as expected and to detect potential problems with the program.

#### Assessment of the initial online test version and autoverification system

Subsequently, the initial online test version program was run in a real-life environment to verify the actual validation in May 2017. In the test version phase, the autoverification decision rules were programmed in other computers and the autoverification reports were not sent to the HIS. Meanwhile, a laboratory technologist and a skilled laboratory specialist (professor in the core clinical laboratory) manually verified and revised all routine cases and then released reports to HIS as before. Comparisons were accomplished as follows: the number of samples, cases that passed the autoverification, cases that failed the autoverification, the true-positive cases (intercepted problematic reports), the true-negative cases (auto-released correct reports), the false-positive cases (intercepted correct problematic reports), the false-negative cases (auto-released problematic reports), the sensitivity, the specificity, and the autoverification passing rate. After initial online testing for 1 month, false-negative cases were found due to a tiny or partial clot that was not detected by the instrument. Thus, logical rules were modified to eliminate the false-negative rate in June 2017. Finally, after running and modifying the test version, the false-negative rate was zero, and the program was formally connected online with the LIS in July 2017. The results of all of the samples that passed all the autoverification decision rules would be directly released from the LIS to the HIS without manual verification.

### Assessment of the efficiency of the autoverification system

In the current study, TAT was measured by two means, namely, TAT 1 and TAT 2. TAT 1 is defined as the interval from the sample receipt by the laboratory to the release of the results on the LIS; TAT 2 is defined as the interval from the complete analysis by the instruments to the report verification on LIS. Both TAT 1 and TAT 2 were compared with the corresponding previous months.

### Statistical analysis

Data were analyzed using IBM SPSS statistics software, version 23.0 (SPSS Inc., Chicago, USA). Mann-Whitney U test was used for independent sample analysis. A *P* < 0.05 was considered significant.

## Results

### Definition of limited range

From January 2016 and December 2016, 157,079 historical test results of coagulation profiles were collected. Distribution intervals of clinical large-scale data of coagulation, such as the mean, standard deviation, median, and 5 and 95% percentiles, are shown in Table 1. In this study, we defined the limited range according the intervals of clinical reference intervals, critical range, and 5 and 95% percentiles, and the limited range was 11.00–16.30 s for PT, 30.40–46.40 s for APTT, 14.00–21.00 s for TT and 2.00–6.51 g/L for FBG, as shown in Table 2.

**Table 1:** Distribution intervals of clinical large-scale data in coagulation

	n	Mean	SD	Median	Range	Minimum	Maximum	Percentiles	
								5%	95%
PT	157,097	13.60	2.51	13.10	109.20	10.00	119.20	11.80	16.30
APTT	157,097	36.97	5.76	36.10	153.30	20.80	174.10	30.40	46.40
TT	157,097	17.12	4.04	16.80	216.30	13.00	229.30	15.00	19.40
FBG	157,097	3.80	1.35	3.50	12.94	0.60	13.54	2.21	6.51

*SD* Standard deviation, *PT* Prothrombin time, *APTT* Activated partial thromboplastin time, *TT* Thrombin time, *FBG* Fibrinogen

**Table 2:** Clinical reference intervals, critical range, 5 and 95% percentiles, and the limited range

	Clinical reference intervals	Critical value	Percentiles (5 and 95%)	Limited Range
PT	11.00–14.30 s	≤ 9.00 s or ≥ 70.00 s	11.80–16.30 s	11.00–16.30 s
APTT	32.00–43.00 s	≤ 15.00 s or ≥ 100.00 s	30.40–46.40 s	30.40–46.40 s
TT	14.0 0–21.00 s	> 150.00 s	15.00–19.40 s	14.00–21.00 s
FBG	2.00–4.00 g/L	< 1.00 g/L	2.21–6.51 s	2.00–6.51 g/L

*PT* Prothrombin time, *APTT* Activated partial thromboplastin time, *TT* Thrombin time, *FBG* Fibrinogen

### Validation of the autoverification system

#### Validation of historical records

In historical data analysis, we evaluated 246 items of historical special abnormal data with high or low concentrations. After running through the autoverification protocols and comparing with the historical reports, all items ended in stopping autoverification and none ended in autoverification. Of the 37,821 historical reports of coagulation profiles from January 2017 to March 2017, 29,165 samples passed the autoverification protocols, indicating that the overall passing rate was 77.11%.

#### Validation of initial online test

The autoverification passing rate, number of samples, cases that passed the autoverification, cases that failed the autoverification, true-positive cases, true-negative cases, false-positive cases, false-negative cases, sensitivity and specificity in the real-life online test are shown in Table 3. Because coagulation requisition forms in our hospital usually contained PT, APTT, FBG and TT simultaneously, we evaluated the overall passing rate of the four tests as a whole sample. The overall autoverification passing rate for all of the samples was 77.63% (10,828/13,949) in May 2017. There were 5 cases that the two reviewers decided to stop for manual verification; however, the autoverification passed. These cases were considered a partial or tiny clot, characterized by PT ≥ 14.00 s or APTT ≥ 43.00 s and FBG ≤ 2.00 g/L, despite being within limited range. After modifying the protocols with logical rules, no false-negative cases occurred, and the passing rate was 78.75% (11,257/14,295) in June 2017.

**Table 3:** Validation of the initial online test

	May 2017	June 2017
n	13,949	14,295
Pass	10,828	11,257
Fail	3121	3038
True-positive	858	892
True-negative	10,823	11,257
False-positive	2263	2146
False-negative	5	0
Sensitivity	99.42%	100%
Specificity	82.71%	83.99%
Passing rate	77.63%	78.75%

True-positive, intercepted problematic reports; True-negative, auto-released correct reports; False-positive, intercepted correct problematic reports; False-negative, auto-released problematic reports

#### Validation of the autoverification system

In July 2017, the autoverification was formally introduced, and we observed 83,699 samples from July 2017 to December 2017 to assess the validation. No cases failed the QC check, and approximately 120 items failed analytical error flags per month. Rules of critical value, limited range and delta check were determined by single tests, and the autoverification passing rate is shown in Table 4. After modifying the logical rules, the average overall passing rate was 78.86%, and the 95% confidence interval (CI) for the overall passing rate was [78.25, 79.59%]. To compare with other studies, the passing rates of different assays and systems (commercial tools, middleware or LIS) are shown in Table 5.

**Table 4:** Passing rate of the autoverification system of single tests and whole samples

	n	Autoverification results	PT	APTT	TT	FBG	Whole sample
July 2017	13,416	Pass	12,485	12,389	12,536	12,557	10,453
		Fail	931	1027	880	859	2963
		Passing rate (%)	93.06	92.34	93.44	93.60	77.91
August 2017	14,477	Pass	13,349	13,413	13,723	13,573	11,354
		Fail	1128	1064	754	904	3123
		Passing rate (%)	92.21	92.65	94.79	93.76	78.43
September 2017	13,673	Pass	12,784	12,456	12,585	12,932	10,740
		Fail	889	1217	1088	741	2933
		Passing rate (%)	93.50	91.10	92.04	94.58	78.55
October 2017	13,004	Pass	12,235	12,059	11,989	12,304	10,289
		Fail	769	945	1015	700	2715
		Passing rate (%)	94.09	92.73	92.19	94.62	79.12
November 2017	14,866	Pass	13,832	13,905	14,203	13,857	11,934
		Fail	1034	961	663	1009	2932
		Passing rate (%)	93.04	93.54	95.54	93.21	80.28
December 2017	14,263	Pass	12,995	13,378	13,254	13,157	11,258
		Fail	1268	885	1009	1106	3005
		Passing rate (%)	91.11	93.80	92.93	92.25	78.93

*PT* Prothrombin time, *APTT* Activated partial thromboplastin time, *TT* Thrombin time, *FBG* Fibrinogen

**Table 5:** Comparision of different autoverification systems in the studies

Study	Year	Country	Assay	System Type	Sample Number	Passing Rate
Randell EW et al [6]	2018	Canada	Clinical Chemistry and immunoassay	Middleware	80,867	91.1 to 94.7%
Xia LY et al [27]	2017	China	Clinical Chemistry and immunoassay	LIS	31,349	74.0%
Krasowski MD et al [28]	2014	USA	Clinical Chemistry and immunoassay	Middleware	–	99.5%
Palmieri R et al [29]	2018	Italy	Urinalysis	AutionMAX-SediMAX	1002	52.4%
Sediq AM et al [5]	2014	Egypt	Thyroid Function	LIS	563	63.8%
Zhao Y et al [16]	2014	China	Coagulation	Middleware	2353	82.0%
Our Study	–	China	Coagulation	LIS	83,699	78.86%

*LIS* Laboratory information system

#### Efficiency of the autoverification system

Comparing the TAT from July to December 2017 with that from July to December 2016, for the routine coagulation assay samples, the median of TAT 1 was 126 min before using the autoverification system versus 101 min after processing the autoverification system. The median of TAT 2 was shortened from 41 min to 15 min. Statistically significant differences were observed for TAT 1 and TAT 2 (*P* < 0.001, *P* < 0.001, respectively, Mann-Whitney U test).

## Discussion

Coagulation profiles were chosen for autoverification because coagulation provides essential and timely analytical information and is crucial for patient treatment and evaluation of physical condition. In our hospital, the numbers of routine coagulation profiles, including PT, APTT, TT and FBG, exceeded 12,000 routine samples per month, and autoverification could be the appropriate answer to process the huge workloads and save labour. Until recently, within-laboratory autoverification that integrates into LIS was still relatively rare.

Our study has taken all factors into consideration, including the instruments, standard serum samples, reagents, specimen and logical relationship of the test items. We designed an autoverification system based on a set of rules integrated with the LIS that runs in the following order: QC check, analytical error flags, critical value, limited range check, delta check, logical rules and clinical diagnosis, as shown in Fig. 1. If any of the rules are violated, the outcome will be held up in the LIS for manual verification. QC was the premise of the autoverification, and analytical error flags and critical value performed as the initial screening to avoid basic analytical error.

The key points of the autoverification protocols are building the limited range check, delta check and logical check. The limited range check is vital to samples without historical data, because for those patients delta check is not available. In our laboratory, because the LIS is connected with the HIS, the patient’s diagnosis would be considered first, e.g., if they were undergoing warfarin treatment, the limited range was PT > 40.00 s, and the allowable range for INR was changed. Thus, limited range check that corresponds with patient’s clinical diagnosis or health conditions facilitates the possibility of precise and customized autoverification. Delta check facilitated the technologists to quickly recognize changes in a patient’s status. Patient’s results with historical data failing the limited range but passing the delta check would pass the autoverification. In this circumstance, our LIS-based autoverification can potentially provide verification more precisely than commercial tools or manual verification. Logical rules were applied to enable the technologists to quickly detect the logical relationship between the items and analytical errors.

Our research extends the knowledge into LIS-based autoverification. We defined the limited range based on the large-scale data of 157,079 routine coagulation assays for 1 year, and observed the validation and efficiency of autoverification protocols based on 83,699 samples for half a year to report the validation and benefits of autoverification. The validation of the autoverification rules was assessed through both the historical data and online test version program. When using the historical data for analysis, the autoverification passing rate was 77.28%. During the online test version program, the passing rate was 77.63% in May 2017 and 78.75% in June 2017. We modified the logical rules to minimize the false-negative rate because we believe that the crucial issue of autoverification is to guarantee the utmost medical safety. The average passing rate was 78.86%, and the 95% CI for the overall passing rate was [78.25, 79.59%] when we formally implemented the program. Our results suggest that the decision rules permit the autoverification system to achieve better accuracy and guarantee medical safety with the availability of historical data and diagnosis.

To date, as shown in Table 5, the passing rate of the autoverification system is similar to the results based on other commercial tools or middleware [16, 29], and the passing rate for coagulation in our laboratory is to some extent higher than those of other studies that use autoverification integrated with LIS [5, 27]. Interestingly, Randell EW et al. [6, 8] and Krasowski MD et al. [29] reported higher autoverification passing rate of more than 90%. Well-designed limit range sets and the selective use of delta checks may contribute to the high autoverification passing rates in their researches [6, 8]. Both studies focused on in clinical chemistry and immunoassay tests, whereas our study focused on our coagulation. It is generally accepted that clinical chemistry and immunoassay are common and regular tests for outpatients, inpatients and patients from physical examination centres, while in contrast, coagulation is to some extent specifically prescribed for sick patients in emergency, undergoing anticoagulant therapy or thrombolytic therapy, or patients in pregnancy. Therefore, pre-analytical problems and problematic samples could be the reason for the lower passing rate of coagulation. In addition, our hospital is the perplexing and complicated illnesses treatment center in Northeast China, and many samples from severely ill patients were collected, which may enhance the proportion of problematic samples in coagulation assays.

Compared to other studies that assessed the interobserver degree of agreement between manual verification and autoverification [4, 5], we analyzed the true-positive rate, the true-negative rate, the false-positive cases rate, the false-negative rate, the sensitivity and the specificity. In our study, no false-negative case was observed prior to the high passing rate to ensure that every sample that passes the autoverification system and is auto-released is a correct report, and thus guaranteeing utmost medical safety. This is because higher autoverification passing rates and lower false-positive rates may inevitably bring higher false-negative rates, and thus some problematic samples would be released without attentive manual verification or re-analysis.

When determining the efficiency of autoverification, TAT is the indication most affected by the implementation of autoverification [2]. In the present study, the TAT 1 and TAT 2 were shortened, which is consistent with previous reports [5, 16]. Because the passing autoverification are released immediately to HIS, the TAT is significantly reduced compared with those processed by manual verification, which in turn could save time and decrease the manual workload in clinical laboratories [6]. Furthermore, another benefit is that the autoverification system follows consistent standardized processes, therefore minimizing the potential mistakes made by laboratory technologists due to lack of clinical experience or feelings of fatigue and stress.

This study has limitations to be considered. First, it is difficult to assess the error rate of the autoverification system. Because no obvious mistakes or errors have been reported by patients or clinicians at our hospital for nearly 2 years, we may infer that the autoverification protocols are at least as safe as the previous manual verification. Second, a higher passing rate of the autoverification system should be designed in the future research.

## Conclusions

We developed and assessed an autoverification system for the most commonly used coagulation profile with a set of rules. The autoverification system for coagulation assays based on LIS can halt the samples with abnormal values for manual verification, guarantee medical safety, minimize the requirements for manual work, shorten TAT and raise working efficiency. Furthermore, we desire to design more precise and valuable rules to build a model in which autoverification may best be used on coagulation profiles.

## Data Availability

The datasets used and/or analyzed during the current study are available from the corresponding author on reasonable request.

## Abbreviations

APTT: Activated partial thromboplastin time
AUTO 10-A: Autoverification of Clinical Laboratory Test Result 10-A; Approved Guideline
FBG: Fibrinogen
HIS: Hospital information system
INR: International normalized ratio
LIS: Laboratory information system
PT: Prothrombin time
TAT: Turnaround time
TT: Thrombin time
